# Effects of Applying Different Doses of Selenite to Soil and Foliar at Different Growth Stage on Selenium Content and Yield of Different Oat Varieties

**DOI:** 10.3390/plants11141810

**Published:** 2022-07-08

**Authors:** Shuangnan Hao, Panfeng Liu, Jie Qin, Lifang Song, Wude Yang, Meichen Feng, Meijun Zhang, Chao Wang, Xiaoyan Song

**Affiliations:** College of Agricultural, Shanxi Agricultural University, No. 1, Mingxian South Road, Taigu District, Jinzhong 030810, China; haoshuangnan@163.com (S.H.); liupanfeng2020@126.com (P.L.); klqj112233@163.com (J.Q.); happysonglifang@126.com (L.S.); sxauywd@126.com (W.Y.); fmc101@126.com (M.F.); meijunz@126.com (M.Z.); wcqxx2005@126.com (C.W.)

**Keywords:** sodium selenite, oat, yield, selenium content

## Abstract

(1) Background: With the increase in people’s consumption of processed oat products, the production of selenium (Se)-enriched oat has become a possibility to supplement the human body with Se. Therefore, the effects of various factors on the Se-enriched ability and yield of different oat varieties were comprehensively studied. (2) Methods: cv.“Pinyan 5” and cv.“Bayou 18” were applied at the stem-elongation stage and heading stage in the Jinzhong (JZ), and cv.“Bayou 1” and cv.“Jinyan 18” were applied at the heading stage and flowering stage in the northwestern Shanxi (JXB) with different doses of Na_2_SeO_3_ (0, 5.48, 10.96, 21.92, 43.84, 65.76, 98.64, 0, 5.48, 10.96, 21.92, 43.84, 65.76, 98.64, 147.96 g hm^−2^) by soil application and foliar spraying. (3) Results: The grain Se content and yield of oat were affected by the variety, Se application dose, stage and method of Se supplementation. Additionally, the Se content in oat grain was positively correlated with the Se application dose while the yield of oat first increased and then decreased with the Se application dose. (4) Conclusions: In the JZ and JXB, 21.92 g hm^−2^ and 43.84 g hm^−2^ Se was sprayed on the leaves of cv.“Bayou 18” and cv.“Bayou 1” at the heading stage, respectively, was the most effective Se biofortification program.

## 1. Introduction

### 1.1. Background

Se is a multifunctional life nutrient for humans and is known as the “king of anticancer” among trace elements [1,2]. The recommended daily intake of Se in the United States is 55 μg, and the standard in the United Kingdom is 75 μg d^−1^ for men and 60 μg d^−1^ for women [3]. The Chinese Nutrition Society recommends that the normal adult intake of Se is 50–250 μg d^−1^ [4], but more than 40 countries and ecological regions in the world are severely Se deficient, of which 72% of ecological regions in China belong to Se-deficient or low-Se ecological regions, resulting in approximately 2/3 of China’s population having varying degrees of insufficient Se intake [5]. Se deficiency in the human body can cause various diseases, such as Kashin–Beck disease, Keshan disease and cardiovascular and cerebrovascular diseases [2,6]. By supplementation with exogenous Se, inorganic Se can be transformed into an organic state that is beneficial to the human body during growth and crop development. For example, in this way, selenite inhibits the entrance of viruses into healthy cells, decreasing (COVID-19) infectivity and reducing mortality. [7] In the food chain, crops are the main source of Se absorption by the human body [8], and it is also the safest and most effective way for humans to absorb Se. For humans, food crops are widely consumed, and they are more suitable for Se bioaugmentation. Therefore, increasing the Se content of crops through agronomic measures will be an important way to effectively improve the Se nutritional status of the human body.

Oats are widely planted feed, forage and food crop in the world and are known as the world’s “third staple food” [9]. Its protein and crude fat contents and quality are higher than those of wheat, rice, corn and other crops [10]. Oats are rich in essential amino acids, dietary fiber, and β-glucan and especially contain oat alkaloids and saponins, which are not found in other grains [11]. Oat also has physiological functions, such as lowering blood sugar, lowering blood lipids, antioxidation and anticancer activity [12]. Shanxi Province has a long history of small grain cultivation. The oat production efficiency index in the JZ ecological region is relatively high. The JXB ecological region is an important oat production base in Shanxi Province and even the whole country. Therefore, the study of Se-enriched oat in the JZ and JXB ecological regions is of great significance to the development of the Se-enriched grain agricultural industry, the improvement of grain quality and the increase in farmers’ income.

### 1.2. Research Progress

At present, the effects of sodium selenite and sodium selenate on the yield and Se content of crops [13], vegetables [14], and fruits [15] through Se application of soil and foliar spraying have been reported. Previous research found that an appropriate amount of exogenous Se can increase oat yield and grain Se content [16]. Studies have shown that spraying Se on different varieties of oat at various growth stages can increase the Se content in grain, but the increase is affected by the variety and the Se dose [17]. Studies have also shown that, compared with soil application and spraying with Se fertilizer, soil application + spraying can increase the yield of oat [13]. The Se content of oat has been increased by supplementing oat in different ecological regions in Finland, but there were large differences in each ecological region [18].

### 1.3. Research Entry Point

Previous studies mainly focused on the effects of one or several factors among varieties, Se application dose, stage and methods of Se supplementation, as well as different ecological regions on the yield or grain Se content of oat. Comprehensive consideration of the effects of various factors on oat yield and grain Se content in different ecological regions is still unclear. Therefore, in the two ecological regions of the JZ and JXB, this study adopted an orthogonal design to study the comprehensive effects of different Se application doses, methods of Se supplementation and application stages on the yield and grain Se content of four oat varieties. This was expected to provide a theoretical basis for the safe production of high-yield Se-enriched oat.

## 2. Results

### 2.1. Effects of Various Factors on Oat Yield

#### 2.1.1. Effects of Various Factors on Oat Yield in the JZ Ecological Region

The order of the influence of various factors on the yield was B (variety) > A (Se dose) > D method of Se supplementation) > C (stage of Se supplementation). The variety and Se dose have a greater impact on the yield of oat. When the Se dose increased, oat yield first increased and then decreased, and the “Bayou 18” was higher than that of “Pinyan 5”. Therefore, considering individual effects, when the foliar spray dose was 65.76 g hm^−2^ Na_2_SeO_3_ on the “Bayou 18” at the heading stage, the oat yield was the highest (Table 1).

Using the extremely significant factors on oat yield, that is, Se dose and variety, to construct an oat yield prediction model, the results were as follows:

Y = −0.007A^2^ + 1.079A + 607.012B + 1173.492 (1)

In the formula, A was the Se dose, B was the variety; the model correlation coefficient r = 0.730, the regression model F value was 7.406, and the regression was significant (Table 2). Oat yield in the JZ ecological region was predicted in the experiment. Calculating the extreme value of formula (1), A = 77.071, and B was variety 2; that is, when a Se dose of 77.071 g hm^−2^ was applied to the “Bayou 18”, the oat yield reached the highest.

#### 2.1.2. Effects of Various Factors on Oat Yield in the JXB Ecological Region

Through a comprehensive analysis of four factors, as the Se dose increased, oat yield first increased and then decreased, and the “Jinyan 18” was higher than that of “Bayou 1”. When the foliar spray dose was 43.84 g hm^−2^ Na_2_SeO_3_ on the “Jinyan 18” at the heading stage, the oat yield was the highest (Table 3).

Using the extremely significant factors on oat yield, that is, Se dose and variety, to construct an oat yield prediction model, the results were as follows:

Y = −0.084A^2^ + 10.004A + 413.708B + 2715.469 (2)

The model correlation coefficient of the model was r = 0.766, the F value of the regression model was 9.23, and the regression was significant (Table 2). Oat production was predicted in the experiment. When a Se dose of 59.548 g hm^−2^ was applied to the “Jinyan 18”, the oat yield reached the highest.

### 2.2. Effects of Various Factors on Se Content in Oat Grain

#### 2.2.1. Effects of Various Factors on Se Content in Oat Grain in the JZ Ecological Region

The effects of Se application dose on the Se content of oat grain reached an extremely significant level, and the varieties reached a significant level. When the foliar spray dose was 147.96 g hm^−2^ Na_2_SeO_3_ on the “Bayou 18” at the heading stage, the Se content in oat grain was the highest (Table 4).

Using the Se dose and the variety, to construct an oat grain Se content prediction model, the results were as follows:

Y = 0.003A + 0.030B + 0.147(3)

The model correlation coefficient was r = 0.966, the F value was 90.073, and the regression was significant (Table 5). The formula can be used to predict the Se content of oat grain. According to the national food security Se-enrichment standard of China, the maximum Se content in grain was Y = 0.300. When Se dose of 41 g hm^−2^ was applied to the “Pinyan 5” or 31 g hm^−2^ was applied to “Bayou 18”, the Se content of oat grain was within the national safety Se-enriched standard of China reach the highest within the range.

#### 2.2.2. Effects of Various Factors on Se Content in Oat Grain in the JXB Ecological Region

The effects of Se application dose and variety on the Se content of oat grain reached extremely significant levels. When the foliar spray dose was 147.96 g hm^−2^ Na_2_SeO_3_ on “Bayou 1” at the heading stage, the Se content in oat grain was the highest (Table 6).

Using the factors on the Se content of oat grain, that is, the regression model of Se dose and cultivar to construct Se content in oat grain was as follows:

Y = 0.003A − 0.086B + 0.250(4)

The model correlation coefficient was r = 0.980, and the F value was 161.466, which can be used to predict the Se content in oat grain (Table 5). According to the national food security Se-enrichment standard of China, the maximum Se content in grain was Y = 0.300. When an Se dose of 45.33 g hm^−2^ was applied to the “Bayou 1” or 74 g hm^−2^ was applied to the “Jinyan 18”, the Se content of oat grain was within the national safety Se-enriched standard reached the highest within the range.

### 2.3. Effects of Various Factors on Oat Yield and Grain Se Content in Separate Ecological Regions

#### 2.3.1. Effects of Various Factors on Oat Yield in Different Ecological Regions

Comprehensive analysis of various factors’ effects on oat yield in the two ecological regions showed that Se dose and variety had a greater influence on oat yield, and foliar spraying at the heading stage had the best effect.

In the JZ ecological region, when the Se dose was 65.76 g hm^−2^, the oat yield reached the highest, and the “Bayou 18” yield was higher than that of the “Pinyan 5”. In the JXB ecological region, when the Se dose was 43.84 g hm^−2^, the oat yield attained the highest levels, and the “Jiyan 18” yield was higher than that of “Bayou 1”.

#### 2.3.2. Effects of Various Factors on Se Content in Oat Grain in Different Ecological Regions

Through the comprehensive analysis of the Se content of oat grain in different ecological regions, it can be seen that Se dose and variety had a greater impact on the Se content of oat grain. Under the same Se dose, the Se content in oat grain, “Bayou 18” was better than that of “Pinyan 5” in the JZ ecological region. The Se content in grain of “Bayou 1” was better than that of “Jinyan 18” in the JXB ecological region, and the effects of foliar spraying at the heading stage among the four varieties were the best.

## 3. Discussion

### 3.1. Effect of Se Dose on Oat Yield and Grain Se Content

According to RCT (randomized control trials) evaluation, in the long run, eating approximately 100 g of oats per day for adults is the most beneficial to human health [19]. At the same time, combined with the recommended daily Se intake standards for adults in different countries and the production of safe Se-enriched food, high-yield Se-enriched crops should consider the grain Se content and yield.

The effect of Se on plant growth and development conforms to the well-known Shelford’s law of tolerance; that is, when the Se dose is low, it can promote plant growth, and if it is too high, it will be toxic to plants [20]. A study found that an appropriate amount of Se can increase oat yield. Under the same Se application conditions, oat yield in the JZ ecological region and JXB ecological region showed a trend of first increasing and then decreasing with the increase in the amount of Se, and similar results were obtained in previous studies on buckwheat [21]. At the same time, studies on the effects of exogenous Se on gramineous crops such as wheat [22] have been confirmed.

The addition of exogenous Se can effectively increase the crop grain Se content. Research showed that when oats were treated with different doses of exogenous Se, the Se content of the grain increased with increasing Se dose [13]. This study demonstrated that the dose of exogenous Se was positively correlated with the Se content in oat grain, which was consistent with previous studies. As a processed product for human consumption, it was not that the higher the Se content in the grain was, the better. The oat Se dose in the JZ ecological region exceeded the Chinese safe Se-enrichment standard (0.10–0.30 mg·kg^−1^) when the Se dose was 147.96 g hm^−2^, which cannot be used as a standard for determining the amount of exogenous Se.

### 3.2. Effect of Se Application Stage on Oat Yield and Grain Se Content

There are some differences in the absorption and transformation efficiency of exogenous Se in different growth stages of plants. Applying Se to wheat in the flowering stage of wheat is more conducive to an increase in yield [23], and even under the premise of water stress, spraying Se during corn vegetative growth and the dough stage can increase corn yield [24]. In this study, oats were treated at various growth stages in the JZ ecological region and JXB ecological region, showing that the treatment effect was best when oats were treated in the heading stage in both ecological regions. This may be due to the transfer of photosynthetic products to the ear during the plant reproductive growth stage, which was also the best time for the ear to absorb Se.

Se application at different growth stages will cause plants to absorb Se differently. For example, studies have shown that foliar spraying of sodium selenite during the reproductive stage of rice has the highest Se content in the grain [25], and others have reported that the Se content in wheat grain was the highest when Se was applied in the early filling stage [26]. This study found that oats in two ecological regions had the best Se content at the heading stage, which was consistent with previous research results. This may be because the heading stage was the stage of oats’ vigorous reproductive growth. At this time, applying Se to oats had a higher absorption and operation efficiency, which can transfer exogenous Se applied to the ears faster.

### 3.3. Effect of Se Application on Oat Yield and Grain Se Content

At present, the main agronomic measures for Se bioenhancement are Se application in the soil and applying to the leaves [27]. Generally, exogenous Se is applied to crops in the form of selenite or selenate, and the crop uptake and conversion rate are high. This study found that in two ecological regions, foliar Se spraying and soil Se application for different oat varieties would increase oat yield within a certain range. This was because the utilization rate of Se first increased and then decreased with increasing exogenous Se dose. Excessive exogenous Se doses have a toxic effect on oat plants, and the utilization rate of Se decreases [20]. This study also found that applying exogenous Se to the same variety in the same ecological region has a better effect than spraying Se on the leaves because the assimilation of plant Se mainly occurs in aerial parts [28] and mainly occurs in leaf chloroplasts [29].

Various methods of Se supplementation lead to different plant transport and absorption efficiencies. In both foliar Se spraying and soil Se application, the Se transfer coefficient increased with increasing exogenous Se dose. Compared with applying Se to the soil, the bioavailability of foliar spraying Se was higher [30]. Similar results were obtained in previous studies on wheat [22]. This may be because selenite is easily combined and fixed by carbonate and iron and manganese oxides after entering the soil, and it is difficult to be directly absorbed and utilized by plants in large quantities [16].

### 3.4. Effect of Abiotic Factors in Different Ecological Regions on Oat Yield and Grain Se Content

Various ecological environments in different ecological regions will also have a certain impact on quality [31]. Research has reported that the efficiency of winter wheat agronomic bioaugmentation is affected not only by the amount of Se applied and the method of Se application but also by the climatic conditions and soil environment of the ecological region [22]. In this study, the oat yield in two different ecological regions increased within a certain Se dose range, but the increase was different. This further proves that crop yield and grain Se content were affected by abiotic factors.

Abiotic factors in different ecological regions, such as soil Se content, soil quality, soil environment and climate environments, such as temperature and precipitation, have certain differences, which have a great influence on crop growth. For example, for oat grown in different ecological regions in Finland, there was a correlation between the Se content in the soil and the grain content [18]. Additionally, studies have shown that there was an extremely significant positive correlation between the Se content in oat grain and soil organic matter [32] In this study, the Se content of oat grain in both the JZ ecological region and JXB ecological region increased with the increase in Se application dose, but there were some differences in the increased range, which was consistent with previous studies. This was because, on the one hand, oats may be affected by the soil Se content and organic matter content in the ecological region. Among them, at lower Se levels, the Se content of JZ (total Se in soil, 0.21 mg kg^−1^; organic matter, 18.96 g kg^−1^) oat grain is higher than that of JXB (total Se in soil, 0.09 mg kg^−1^; organic matter, 95.35 g kg^−1^). On the other hand, when the roots absorb and transport SeO_3_^−^^2^ and SeO_4_^−^^2^, the roots absorb part of it, while the other part is transported to chloroplasts and assimilated into organic Se [33]. It is possible that the original SeO_3_^−^^2^ and SeO_4_^−^^2^ in the soil also participated in transportation to plant leaves, which needs further study.

## 4. Material and Methods

### 4.1. Test Material

Seeds of cv. “Pinyan 5”, cv. “Bayou 18”, cv. “Bayou 1” and cv. “Jinyan 18” were provided by the Institute of Crop Variety Resources, Shanxi Academy of Agricultural Sciences. Exogenous Se is sodium selenite (Na_2_SeO_3_). (Since the organic conversion rate of selenate to selenium is significantly lower than that of selenite, Na_2_SeO_3_ was selected in the experiment.)

### 4.2. Field Experimental Sites

Field experiments were conducted at two experimental sites from May to September 2018: Shenfeng village (37°25′ N, 112°35′ E, elevation 793 m) in Taigu County, Jinzhong City (JZ) and Linjiabao village (39°41′ N, 112°25′ E, elevation 1516 m) in Youyu County (JXB), Shuozhou City, Shanxi Province, China. The climates in the JZ and JXB were warm temperate continental climates and temperate continental monsoon climates, respectively. The annual average temperature in the JZ and JXB were 11.5 °C and 4.98, respectively, annual sunshine hours were 2701.3 h and 2725.4 h, respectively, annual precipitation was 432.4 mm and 597.7 mm, respectively, frost-free stage was 176 d and 104 d, respectively, and average temperature in oat growing stage (May-September) was 669 °C and 525.2 °C, respectively. The soil types of the JZ and JXB test sites were gray cinnamon soil and chestnut soil, respectively. In the 0–20 cm soil layers, the following physical and chemical properties were observed in the JZ and JXB: pH, 7.68 and 7.73; organic matter, 18.96 g kg^−1^ and 7.66 g kg^−1^; total nitrogen, 2.78 and 0.74 g kg^−1^; available phosphorus, 10.71 mg kg^−1^ and 6.50 mg kg^−1^; available potassium, 198.52 mg kg^−1^ and 95.35 mg kg^−1^; and total Se 0.21 mg kg^−1^ and 0.09 mg kg^−1^, respectively. Before planting, 150 kg hm^−2^ and 300 kg hm^−2^ compound fertilizers (15% N, 15% P, and 15% K) were applied, respectively.

### 4.3. Experimental Design

The experiment adopted an orthogonal design (Table 7), and 8 Se levels were set: 0, 5.48, 10.96, 21.92, 43.84, 65.76, 98.64 and 147.96 g hm^−2^. In the JZ, two varieties, “Pinyan 5” and “Bayou 18”, were treated with exogenous Se at the stem-elongation stage and heading stage, respectively. In the JXB, two varieties, “Bayou 1” and “Jinyan 18”, were treated at the heading stage and flowering stage, respectively. Two methods of Se application were used in the two test sites: foliar spraying and soil application. When foliar spraying, to facilitate the adhesion and absorption of the leaves, the sprayer was adjusted into a fine mist. The JZ and JXB plot areas were 12 m^2^ and 10 m^2^, and the solution applied was 1.2 L per plot (12 m^2^) and 1 L per plot (10 m^2^), respectively, repeated three times, each with 48 plots. The sowing amount was 172.5 kg hm^−2^, the method of artificial ditching was adopted, and the sowing depth was 4–6 cm. After sowing, the soil was covered and suppressed. Other cultivation management was the same as that of the field.

### 4.4. Measurement Items and Methods

#### 4.4.1. Yield Determination

After oat maturity, 1 m^2^ of uniform growth was selected in each plot to measure the yield.

#### 4.4.2. Determination of Total Se Content in Grain

The oat grain total Se content was determined by hydride atomic fluorescence spectrometry [34]. The specific method was as follows: weighed 0.2 g (accurate to 0.001 g) of ground oat grain powder into the digestion tube, added 10 mL nitric acid and 2 mL hydrogen peroxide, shook and mixed well, and digested in a microwave digester. After the digestion was finished and cooled, a few glass beads were added, and heated was continued on the graphite digestion apparatus until it was nearly dry. Added 5 mL hydrochloric acid solution (6 mol L^−1^), continue heated until the solution becomes clear and colorless with white smoke, cool, transfered it to a 10 mL volumetric flask, added 2.5 mL potassium ferricyanide solution (100 g L^−1^), constant volume with water, mixed well to be tested, and performed reagent blank test at the same time. Measurement by AFS-230E dual-channel atomic fluorescence spectrometry (manufacturer: Beijing Haiguang Instrument Co., Ltd.; the manufacturer headquarters: Zhongguancun, Haidian district, Beijing, China), and the result was expressed as mg kg^−1^.

### 4.5. Data Analysis

Microsoft Excel 2010 was used for data processing, and SPSS 23.0 (IBM Crop., Armonk, NY, USA)was used for variance analysis and regression modeling. Using the significant and extremely significant factors in the variance analysis results, combined with intuitive analysis results, virtual variables [35] are introduced to replace unquantifiable factors to establish the fitting model.

## 5. Conclusions

The effect of Se concentration on oat yield and grain Se content reached an extremely significant level; the effect of oat varieties on yield reached an extremely significant level, and the effect on grain Se content reached a significant or extremely significant level; the effects of Se application period and method on oat yield and grain Se content were not significant. From intuitive analysis and regression modeling, it can be seen that in the ecological regions of the JZ and JXB, it is better to spray sodium selenite on oat leaves at the heading stage, but when the yield reaches the highest and the grain Se content is safe in China. When the Se-enriched standard range (0.10–0.30 mg kg^−1^) reaches the maximum, different varieties of oat have different responses to Se concentration. Considering oat yield and grain Se content, under the condition that the grain Se content meets China’s safe Se-enrichment standard, heading dates of cv. “Bayou 18” and cv. “Bayou 1” in the JZ ecological region and JXB ecological region were considered, and foliar spraying of 21.92 g hm^−2^ and 43.84 g hm^−2^ Na_2_SeO_3_ was the best Se bioenhancement program.

## Figures and Tables

**Table 1:** Effects of various factors on the yield of oat in the JZ ecological region.

Test Number	Se Dose (g hm^−2^) A	Variety B	Stage of Se Supplementation C	Method of Se Supplementation D	Yield (kg hm^−2^)
1	0	1 (Pinyan 5)	1 (Stem-elongation stage)	1 (Soil application)	1003.59
2	0	2 (Bayou 18)	2 (Heading stage)	2 (Foliar spraying)	1650.00
3	5.48	1	1	1	1181.60
4	5.48	2	2	2	1739.06
5	10.96	1	1	2	1321.00
6	10.96	2	2	1	1837.35
7	21.92	1	1	2	1201.70
8	21.92	2	2	1	1747.50
9	43.84	1	2	1	1511.10
10	43.84	2	1	2	2272.39
11	65.76	1	2	1	1576.87
12	65.76	2	1	2	2330.55
13	98.64	1	2	2	1499.50
14	98.64	2	1	1	1775.70
15	147.96	1	2	2	1241.10
16	147.96	2	1	1	1697.00
K1	2653.59	10,536.45	12,783.52	12,330.70	
K2	2920.66	15,049.55	12,802.48	13,255.30	
K3	3158.35				
K4	2949.20				
K5	3783.49				
K6	3907.42				
K7	3275.20				
K8	2938.10				
k1	1326.79	1317.06	1597.94	1541.34	
k2	1460.33	1881.19	1600.31	1656.91	
k3	1579.18				
k4	1474.60				
k5	1891.74				
k6	1953.71				
k7	1637.60				
k8	1469.05				
Range(R)	484.66	564.14	2.37	115.57	
Adjust(R’)	285.41	693.73	2.91	142.12	
Factor priority order		B > A > D > C			
Optimal combination	A6	B2	C2	D2	
ANVOA					
*p* value	0.000	0.000	0.971	0.083	

Note: K, the sum of the experimental indicators corresponding to the factors and levels. k, average of K. For example, K_A1_ = test number 1 and 2 (yield values corresponding), k, average of K. Range (R), represents the difference between the maximum value and the minimum value. Adjust (R’), Converted according to the number of factor levels. *p* value, represents a significant difference at the 0.05 level. The same as below.

**Table 2:** Significance test of regression model of oat yield.

Region	F Value	*p* Value
JZ	7.406	0.007
JXB	9.230	0.003

**Table 3:** Effects of various factors on the yield of oat in the JXB ecological region.

Test Number	Se Dose (g hm^−2^) A	Variety B	Stage of Se Supplementation C	Method of Se Supplementation D	Yield (kg hm^−2^)
1	0	1 (Bayou 1)	1 (Heading stage)	1 (Soil application)	2740.05
2	0	2 (Jinyan 18)	2 (Flowering stage)	2 (Foliar spraying)	3412.80
3	5.48	1	1	1	3200.40
4	5.48	2	2	2	3509.90
5	10.96	1	1	2	3347.27
6	10.96	2	2	1	3663.73
7	21.92	1	1	2	3475.70
8	21.92	2	2	1	3870.20
9	43.84	1	2	1	3922.35
10	43.84	2	1	2	4151.90
11	65.76	1	2	1	3175.60
12	65.76	2	1	2	3734.00
13	98.64	1	2	2	3004.63
14	98.64	2	1	1	3382.83
15	147.96	1	2	2	2875.20
16	147.96	2	1	1	3325.50
K1	6152.85	25,741.20	27,522.60	27,280.67	
K2	6710.30	29,050.87	27,269.47	27,511.40	
K3	7011.00				
K4	7345.90				
K5	8074.25				
K6	6909.60				
K7	6387.47				
K8	6200.70				
k1	3076.43	3217.65	3440.33	3410.08	
k2	3355.15	3631.36	3408.68	3438.93	
k3	3505.50				
k4	3672.95				
k5	4037.13				
k6	3454.80				
k7	3193.73				
k8	3100.35				
Range(R)	960.70	413.71	31.64	28.84	
Adjust(R’)	565.74	508.75	38.91	35.47	
Factor priority order	A > B > C > D				
Optimal combination	A5	B2	C1	D2	
ANVOA					
*p* value	0.000	0.000	0.925	0.777	

**Table 4:** Effects of various factors on Se content in oat grain in the JZ ecological region.

Test Number	Se Dose (g hm^−2^) A	Variety B	Stage of Se Supplementation C	Method of Se Supplementation D	Se Content (mg kg^−1^)
1	0	1 (Pinyan 5)	1 (stem-elongation stage)	1 (Soil application)	0.13
2	0	2 (Bayou 18)	2 (Heading stage)	2 (Foliar spraying)	0.17
3	5.48	1	1	1	0.15
4	5.48	2	2	2	0.20
5	10.96	1	1	2	0.20
6	10.96	2	2	1	0.24
7	21.92	1	1	2	0.25
8	21.92	2	2	1	0.27
9	43.84	1	2	1	0.34
10	43.84	2	1	2	0.36
11	65.76	1	2	1	0.44
12	65.76	2	1	2	0.47
13	98.64	1	2	2	0.46
14	98.64	2	1	1	0.55
15	147.96	1	2	2	0.61
16	147.96	2	1	1	0.56
K1	0.30	2.58	2.67	2.68	
K2	0.35	2.82	2.72	2.72	
K3	0.44				
K4	0.52				
K5	0.70				
K6	0.90				
K7	1.01				
K8	1.17				
k1	0.15	0.32	0.33	0.33	
k2	0.17	0.35	0.34	0.34	
k3	0.22				
k4	0.26				
k5	0.35				
k6	0.45				
k7	0.50				
k8	0.58				
Range(R)	0.43	0.03	0.01	0.00	
Adjust(R’)	0.25	0.04	0.01	0.01	
Factor priority order		A > B > D > C			
Optimal combination	A8	B2	C2	D2	
ANVOA					
*p* value	0.000	0.010	0.517	0.627	

**Table 5:** Significance test of regression model of Se content in oat grain.

Region	F Value	*p* Value
JZ	90.073	0.000
JXB	161.466	0.000

**Table 6:** Effects of various factors on Se content in oat grain in the JXB ecological region.

Test Number	Se Dose (g hm^−2^) A	Variety B	Stage of Se Supplementation C	Method of Se Supplementation D	Se Content (mg kg^−1^)
1	0	1 (Bayou 1)	1 (stem-elongation stage)	1 (Soil application)	0.10
2	0	2 (Jinyan 18)	2 (Heading stage)	2 (Foliar spraying)	0.05
3	5.48	1	1	1	0.16
4	5.48	2	2	2	0.07
5	10.96	1	1	2	0.23
6	10.96	2	2	1	0.12
7	21.92	1	1	2	0.26
8	21.92	2	2	1	0.16
9	43.84	1	2	1	0.29
10	43.84	2	1	2	0.21
11	65.76	1	2	1	0.34
12	65.76	2	1	2	0.29
13	98.64	1	2	2	0.42
14	98.64	2	1	1	0.38
15	147.96	1	2	2	0.57
16	147.96	2	1	1	0.46
K1	0.15	2.37	2.09	2.01	
K2	0.23	1.74	2.02	2.10	
K3	0.35				
K4	0.42				
K5	0.50				
K6	0.63				
K7	0.80				
K8	1.03				
k1	0.08	0.30	0.26	0.25	
k2	0.12	0.22	0.25	0.26	
k3	0.18				
k4	0.21				
k5	0.25				
k6	0.32				
k7	0.40				
k8	0.52				
Range (R)	0.44	0.08	0.01	0.01	
Adjust (R’)	0.26	0.10	0.01	0.01	
Factor priority order		A > B > C > D			
Optimal combination	A8	B1	C1	D2	
ANVOA					
*p* value	0.000V	0.000	0.897	0.738	

**Table 7:** L_16_ (8 × 2^3^) orthogonal test scheme.

Test Number	Se Dose (g·hm^−2^) A	Variety B	Stage of Se Supplementation C	Method of Se Supplementation D
1	1(0)	1 (J Z-Pinyan 5)	1 (J Z-stem-elongation stage)	1 (Soil application)
		1 (JXB-Bayou 1)	1 (JXB-Heading stage)	
2	1	2 (J Z-Bayou 18)	2 (J Z-Heading stage)	2 (Foliar spraying)
		2 (JXB-Jinyan 18)	2 (JXB-Flowering stage)	
3	2(5.48)	1	1	1
4	2	2	2	2
5	3(10.96)	1	1	2
6	3	2	2	1
7	4(21.92)	1	1	2
8	4	2	2	1
9	5(43.84)	1	2	1
10	5	2	1	2
11	6(65.76)	1	2	1
12	6	2	1	2
13	7(98.64)	1	2	2
14	7	2	1	1
15	8(147.96)	1	2	2
16	8	2	1	1

Note: L, represents an orthogonal table. In the table, 16, the number “16” in the lower right corner of L indicates that there are 16 rows, and this orthogonal table is used to arrange the experiment to contain 16 treatments. The number 8 means that there is a column with a level number of 8, which means that there are 8 different Se doses of sodium selenite. The 2^3^ means that there are 3 columns with a level number of 2, that is, there are two varieties, two periods of applying sodium selenite and two ways of applying sodium selenite.

## Data Availability

Not applicable.
